# Enhancing the physician-scientist workforce: evaluating a mentored research program for medical students’ research competencies and intentions

**DOI:** 10.21203/rs.3.rs-4830569/v1

**Published:** 2024-08-29

**Authors:** Brooke Piercy, Nicole Miovsky, Harinder Singh, Behnoosh Afghani, Margaret Schneider

**Affiliations:** Institute for Clinical and Translational Science, University of California, Irvine; Institute for Clinical and Translational Science, University of California, Irvine; Institute for Clinical and Translational Science, University of California, Irvine; Institute for Clinical and Translational Science, University of California, Irvine; Institute for Clinical and Translational Science, University of California, Irvine

**Keywords:** mentored research program, medical student research, research competencies, research career intentions, physician-scientist workforce, program evaluation

## Abstract

**Background:**

The growing recognition of the need to incorporate scientific discoveries into healthcare decisions underscores an urgency for a robust physician-scientist workforce to advance translational research. Despite the correlation between medical students’ research engagement and their academic productivity and success, significant gaps remain in the scientific workforce exacerbated by the “leaky pipeline” phenomenon from medical school to academic medicine, where potential physician-scientists veer away from research careers.

The purpose of this study was to assess the effectiveness of a structured mentored research program for enhancing medical students’ research competencies and sustaining their interest in research careers, thereby potentially enhancing the physician-scientist workforce.

**Methods:**

The Medical Student Research Program (MSRP) implemented at the University of California, Irvine (UCI) was designed to provide comprehensive research training and support to medical students through a series of structured lectures, mentorship by dedicated faculty, and administrative support for research activities. Students were surveyed upon enrollment and one year later to assess the change in research competencies from baseline to follow-up (paired samples t-test), students’ intent to use research in clinical practice (paired samples t-test), and their intent to conduct research in the future (McNemar’s test and McNemar Bowker test).

**Results:**

Preliminary evaluations indicated that the MSRP enhanced students’ research competencies and has the potential to enhance medical students’ research skills. However, similar to national trends, there was a decrease in students’ intentions to engage with research in their future clinical career.

**Conclusions:**

Our preliminary findings demonstrate MSRP students’ enhanced research competencies during the first year of the program. However, the decline in students’ intentions to engage in future research highlights the need for continued innovation in research training programs to sustain future intent to conduct research, in turn helping to address the “leaky pipeline” in the physician-scientist workforce. Future studies should focus on mid and long-term outcomes to fully assess research program impact on the physician-scientist pipeline and on integrating such programs more broadly into medical education.

## Background

The continual need to increase the effectiveness of the healthcare system by utilizing advances in scientific discoveries has highlighted the mandate for a more robust translational research workforce pipeline(1). As suggested by both clinicians and researchers, there is an urgent need for programs which foster strong physician-scientist training in basic science, clinical, and population health research with the long-term aim of improving the health of the public(2–7). There are data suggesting that an emphasis on research experience during medical school is correlated with both short and long-term scientific productivity, success in academia, greater instances of publishing first-authored papers(8), and an increase in students’ interest in pursuing research throughout their clinical career(8, 9). However, despite the reported interest of the majority of matriculating medical students in research^8^ and the growing emphasis on research training programs in medical schools, several gaps persist as medical students continue their journey and become faculty physician scientists^7^. Although NIH funding increased between 2016 and 2020, the number of postdoctoral research fellowships awarded to MDs has been far below historic levels over the last four decades(10). Recent trends suggest that a “leaky pipeline” in the physician-scientist workforce is contributing to this problem(11), in which many researchers or trainees who intend to pursue research early in their career ultimately do not end up doing so. Factors contributing to this decline in the physician-scientist workforce include a lack of mentorship opportunities for young investigators, the attrition of women in the field, not identifying physician-scientists at an early enough point in their career to better prepare them for the future, too few opportunities for funding to secure protected research time and resources, the cost of attending medical school, a lack of training opportunities that teach foundations of research, and high clinical demand placed upon early-career clinicians(6).

Compounding the need for programs that encourage and facilitate research among medical students, the United States Medical Licensing Examination (USMLE) STEP 1 exam score reporting to residency programs was revised after January 2022 to be Pass/Fail, as opposed to a numeric report. With this change, we expect that acceptance into residency programs will become even more competitive and that many medical students will be motivated to take on additional extramural activities, including research, to bolster their applications(12, 13). With all these factors at play, incorporating research training programs into the medical curriculum is an important component of preparing clinical trainees for the highest level of success in their medical careers. In fact, in a formal endorsement of such programs, the Liaison Committee on Medical Education (LCME) Standards under section 3.2 require support for research opportunities within the medical education community^19^, however, there is no specification regarding the best ways to implement or address the challenges of incorporating such research training programs into the medical school curriculum. Studies have noted a shortage of effective mentors and lack of time, infrastructure, funding and skills in starting a project as barriers for medical students to undertake research(14, 15).

To address the barriers to engaging in research and to provide increased opportunities for medical students to gain research experience, some universities have created medical student training programs structured to provide research experiences amongst future clinicians(16, 17). Curricula that include exclusive didactic teaching have been shown to be less effective as compared to those that include assigned mentors and span the 4 years of medical school(17). However, according to reviews of the literature(17, 18) only a few medical schools have reported longitudinal training programs that not only teach the fundamentals of research to beginner medical student scientists but also allow them to accumulate experience and develop skills throughout the four years of medical school. These programs have predominantly focused on research mentorship in a specific specialty, such as research in rural communities, integrative or primary care medicine(19, 20) or have been of shorter duration. For example, the University of Texas at Galveston’s Medical Student Research Program has been effective for increasing the interest of participants in engaging in research, but this program is limited to the summer between the first and second year of medical school for the majority of participants(21). The George Washington University School of Medicine and Health Sciences provides the Scholarly Research Concentration; however, this program only provides a standard set of research opportunities with dedicated resources for students spanning 18 months during the pre-clinical MD training(22). The University of Arizona has developed a long-standing four-year research training program for medical students and has enrolled more than 1000 students in research(23), yet no data have been published on the impact of this program on the students.

At the UCI School of Medicine, we introduced a training program that builds on existing research training curricula to motivate research involvement among medical students. Based on the successes reported by other universities(16, 21, 24–28) we developed and piloted the Medical Student Research Program (MSRP), a four-year mentored research program with dedicated research resources and structured support from the UCI Institute for Clinical Translational Science (ICTS), with the long-term goal of improving the health of the public by encouraging medical students to pursue careers as physician-scientists and to enhance their clinical capabilities with advanced knowledge of how research impacts care(18) .

Our objective was to create a program inclusive to all medical students interested in research with the following defining features: 1) a designated home on campus they could turn to for support in finding a dedicated faculty mentor with the time and resources to commit to training; 2) a series of structured lectures focused on research best practices to boost research knowledge and competency, 3) assistance with administrative hurdles such as submitting Institutional Review Board protocols and obtaining extramural funding as well as assistance in overcoming research hurdles and barriers; and 4) periodic follow-up to determine how this structured program impacts research interest and engagement.

As noted in Carberry et al.(18), there is a distinction within medical education training between preparation for “using research” and “doing research,” and for this study we assessed both. In this paper, we describe the components of the UC Irvine School of Medicine MSRP and share the impact of the program on students’ self-perceived gain in research competencies and intention to use research in their future clinical practice (“using research”), as well as their intention to conduct research themselves (“doing research”). We evaluated the impact of the program on these perceived knowledge gains and future intentions to use and engage in research, and to gain a better insight on the program’s impact to strengthen the future physician-scientist workforce.

## Methods

### Study Context

#### The Medical Student Research Program (MSRP)

The MSRP is a four-year research program offered to all entering UCI medical students. The program starts in their first semester and spans the duration of their MD training until graduation. Considerations of medical students’ specific needs and challenges were considered when developing the MSRP. These included: 1) the lack of time medical students have to pursue research, particularly in their third and fourth years when clinical rotations begin and residency interviews are underway; 2) the wide-range of research knowledge that students possess when entering medical school, from little-to-no research experience to several years of laboratory practice; 3) a need to identify faculty mentors in their area of interest; and 4) student unfamiliarity with campus resources to support a research project. We structured our program in a way that specifically addresses these four considerations.

**Figure 1:**
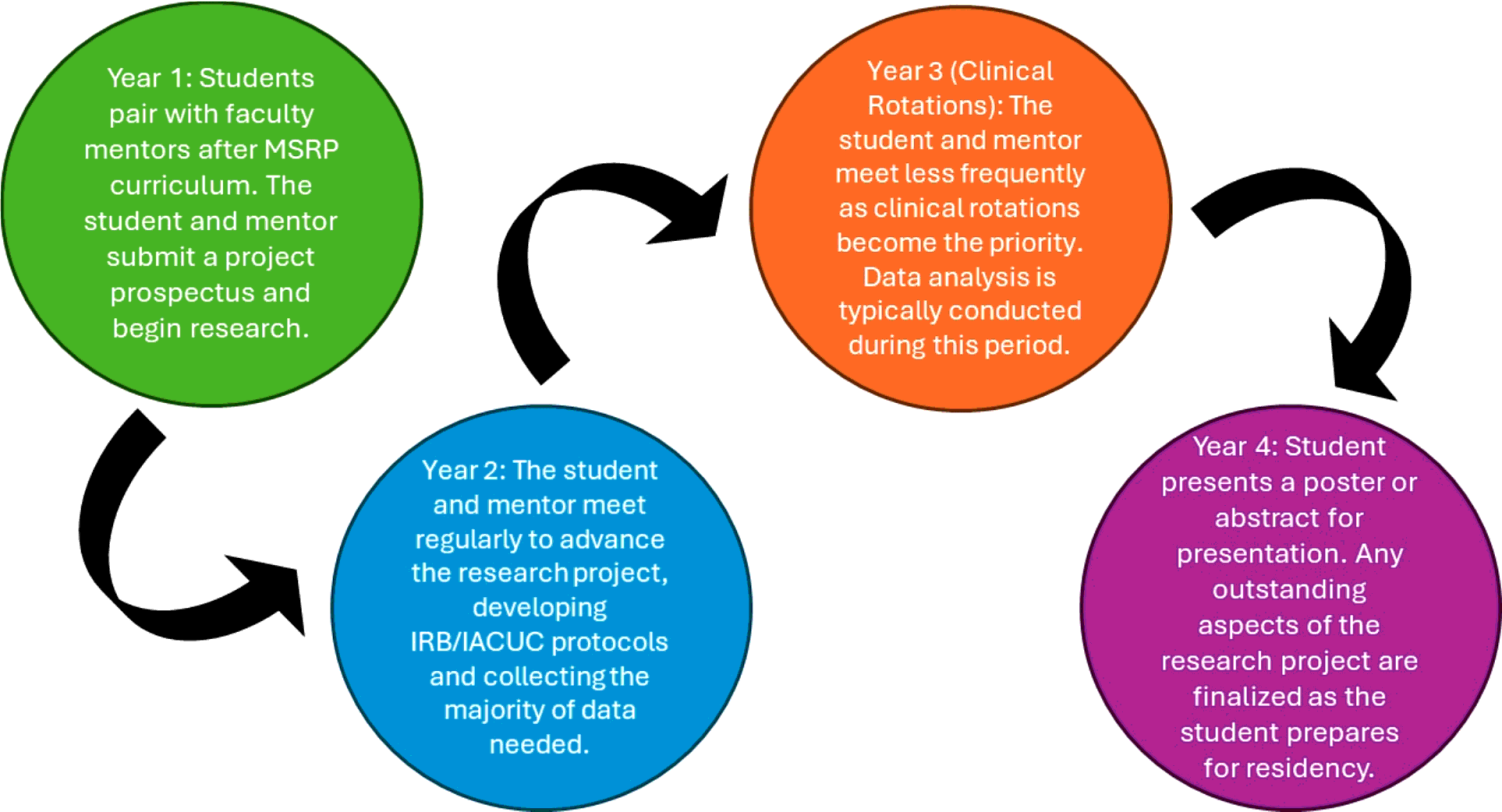
Overview of ICTS MSRP Timeline Across All Four Years of Medical Education

The MSRP responded to these considerations by taking into account the time demand on students over the four years of medical school (Fig. 1). During the first year, when students have relatively more available time for research, students in MSRP attended 10 didactic sessions covering topics including the basics of research methods and statistics, research ethics, professional development for a career in research, and other courses to familiarize students with the fundamentals of conducting research. Also during this first year, students were matched with a faculty mentor who guided and trained the students through one-on-one research experiences spanning basic science to clinical and translational research. The didactic and mentor-matching components of the MSRP were supported administratively by the ICTS at UCI. Students in the MSRP were expected to be maximally engaged in research during the summer after their first year and into their second year of medical school and were encouraged to use the summer between the first and second years to immerse themselves in their research project. Summer research was facilitated by the ICTS which assisted students in applying for research funding. Throughout the program, the ICTS provided support for students in obtaining regulatory approvals (both human and animal research), in writing and submitting manuscripts for publication, and in analyzing and interpreting their data. ICTS staff also met with students individually periodically to assist them in overcoming other hurdles to the research process.

**Figure 2:**
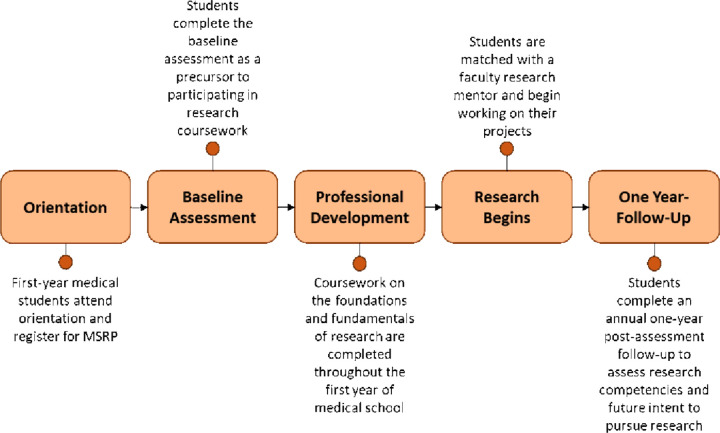
MSRP Timeline of Year 1

An in-depth overview of MSRP Year 1 is provided in Fig. 2. At the beginning of Medical Student Year 1 (MS1), prior to matching with a faculty mentor, students participated in a 10-session didactic lecture series focusing on the foundations and basic elements of conducting research (Table 1). After completing the didactic course, students were paired with a faculty research mentor and spent the next two years designing and implementing a research project that included seeking Institutional Review Board (IRB)/Institutional Animal Care and Use Committee (IACUC) approval as necessary and collecting data. All faculty were made aware of the time constraints of a medical student (as opposed to pre- or postdoctoral trainees) before the partnership was finalized, which helped both parties align expectations about the amount of time that could be spent on the research project. Students were expected to spend at least eight weeks of the summer after completing the MS1 year on their research, and a minimum of two hours per week on research during the academic year. While in dedicated study for the USMLE STEP 1 exam in Medical Student Year 2 (MS2), students paused work on their research and had the option to continue after completing the exam and beginning clinical rotations. In the students’ final 2 years of the program, submission to a scientific conference or meeting was required along with presentation of research results in abstract or poster format.

#### Program Evaluation

MSRP student research competencies and future intentions to engage with research were assessed through self-report surveys. Before entering the program, the majority of students completed a baseline survey to determine their level of research competencies, intent to pursue a career in research, and intent to use research in their clinical practice. After being paired with their faculty mentor and completing their first year in the MSRP, each student completed the survey again.

The data presented within this paper were analyzed after data were available from five cohorts of medical students, who enrolled in the Medical Student Research Program (MSRP) at UCI between 2018 and 2022. Although the program officially began in 2016, evaluation and tracking data were not standardized until two years after program inception. For this reason, students from the 2016 and 2017 cohorts have been excluded from data analysis.

### Procedure

#### Data collection

First year medical students interested in joining MSRP completed a baseline survey online in the fall of their first year. After completing their first year of medical school and prior to beginning of second year, the students were sent a link to a one-year follow-up survey. Those who had not completed the survey received automatic reminders at weekly intervals for 5 weeks.

Study data were collected and managed using Research Electronic Data Capture (REDCap) tools hosted at UCI(29, 30). REDCap is a secure, web-based software platform designed to support data capture for research studies, providing 1) an intuitive interface for validated data capture; 2) audit trails for tracking data manipulation and export procedures; 3) automated export procedures for seamless data downloads to common statistical packages; and 4) procedures for data integration and interoperability with external sources.

#### Survey Measures

Both the baseline and follow-up assessment included items on demographics and student characteristics, research competencies and intentions to engage with research in the future. Questions on research competencies and intent for using research were developed by the Clinical and Translational Science Awards (CTSA) Education Core Competency Working Group and based on the Core Competencies in Clinical and Translational Science(31–33). Competency questions asking about future intent to conduct research were modelled on items from national surveys by the Association of American Medical Colleges (AAMC) that are administered to matriculating students and at multiple times throughout medical school(34, 35).

The MSRP annual survey included a 12-item index of perceived research competencies (see Table 2), and a five-item index of intent for using research in clinical practice (see Table 3). The perceived competencies index had a very high internal consistency (Cronbach’s alpha of 0.92), and the using research index also had strong internal consistency (Cronbach’s alpha of 0.84).

The MSRP baseline and one-year follow-up surveys also included two questions modelled on the AAMC items about a future intent for doing research: “In which of the following activities do you plan to participate during your career?” (with an option to select “Research”) and “How exclusively do you expect to be involved in research?” (with response options “Full-time”, “Significantly involved”, and “Involved in a limited way”).

### Analyses

The Chi-square test of independence, and when appropriate the Fisher-Freeman-Halton exact test, was conducted to assess if non-response at the one-year post-assessment had a significant relationship to race, Hispanic or Latino/Latina ethnicity, or gender.

The analyses of program impact were limited to students who responded at both the baseline and one-year follow-up assessment. Pre-post comparisons were carried out to examine the impact of the MSRP, with paired samples t-tests to compare change over time in research competencies and in intent to use research in clinical practice (“using research”) from baseline to follow-up. Additionally, McNemar’s test and the McNemar-Bowker test were used to examine change over time in student’s future intent to conduct research (“doing research”).

## Results

### Participant Characteristics

Two hundred medical students across five cohorts were enrolled in the MSRP at UCI during the time period of the study. Of those enrollees, 111 students completed both the baseline and one-year follow-up and were therefore included in this analysis (a 56% response rate from the population of MSRP students). The sample (N = 111) was racially and ethnically diverse (43% Asian/Pacific Islander, 38% Caucasian, 10% Hispanic/Latinx) with 6.3% identifying as more than one race and 6.5% not reporting. The distribution of MSRP students’ self-reported gender was 39.4% male, and 60.6% female, closely approximating the UCI School of Medicine gender distribution (57% female) and AAMC national data (54% female)(36). Comparisons between students who responded to the one-year follow-up survey (N = 111) and those who only responded at baseline (n = 59) revealed no significant differences in demographics between the two groups at baseline for gender, race or ethnicity.

### Change in Research Competencies

Students’ self-perceived research competencies increased over time [t(110) = 5.65, p < .001]. The effect size for this increase in competencies was in the range typically considered moderate (d = 0.54), see Table 4.

### Change in Intent for Using Research

Ratings from students on the index for “using research” decreased from baseline to the one-year follow-up [t(110) = −2.54, p = .012], showing a decline in the intent to use research in future clinical practice, with a small effect size of d = −0.24. Table 4 shows test p-values, means, and standard deviations at baseline and follow-up.

### Change in Future Intent to Conduct Research

At baseline, 87.4% of MSRP students reported intending to conduct research in their future career. McNemar’s test (N = 111) was used to compare self-reported intent to conduct research (for “doing research”) at baseline to the one-year follow-up. The test showed a significant drop in the overall proportion of students who intended to pursue any amount of research in the future, from 87.4–78.4% (p = .021).

Similarly, a McNemar-Bowker test (N = 111) showed a significant decrease after one year (p = .023) in how involved students expected to be in research (e.g., either full-time, significantly involved, or involved in a limited way). The trend seems to have been driven by the decreased proportion of those who want to be involved in research in a significant way (55.9% at baseline as compared to 45% at the one-year follow-up). Despite this, students remained interested in some involvement in research, with the proportion of those who said they intend to do research in a limited way changing from 43.2% at baseline to 54.1% at the one-year follow-up. Students who intend to be involved in research full-time stayed the same (1% of respondents).

## Discussion

In this report, we examined the impact of the first year of the MSRP on students’ intentions to engage in research, research competencies, and intentions to use research in clinical practice. The survey data indicated that students’ research competencies increased over time, whereas the proportion of students who intended to conduct research and/or to use research in clinical practice declined.

### Change in Research Competencies

Our results showed MSRP students’ self-perceived research competencies increased over time. Specific items on the competency scale assessed the students’ perceived abilities to engage in a range of activities that comprise the research process (e.g., evaluating published research, formulating a research question, designing a research protocol, etc.). Because these data were collected at the end of the first year of medical school, the impact of research competencies may represent the impact of the 10-session didactic portion of the MSRP, rather than the impact of their involvement in research. At the time of the one-year follow-up assessment survey, students had only been engaged with their research mentors for approximately 6 months, so the impact of the research experience and mentoring component is more likely to emerge with longer-term follow-up. The self-perceived gain in competencies is consistent with previous studies that tracked long-term research productivity among students who completed at least one research year or participated in organized research curriculum(21, 27, 28), and affirms the value that the MSRP adds to the medical school curriculum, especially for those students seeking to combine clinical care with research in their future careers.

### Change in Intent to Use Research in Clinical Practice

This study found a decrease in MSRP students’ intent for using research during clinical practice after one year of being involved in MSRP. While there has been a large emphasis in the medical field to encourage medical student trainees to “do research” rather than “use research” to prevent the decline in the physician-scientist workforce(18), this waning trend of “using research” may be an equally important precursor to the decline in clinician-scientists. While not all clinicians will go on to become physician-scientists and lead their own research teams, aspects of “using research” such as seeking new evidence-based treatments and keeping up with medical literature are critical elements all clinicians should infuse into their practice. A decline in these intentions may be indicative of a disconnect in how important research is to clinical practice even when one is not actively conducting research themselves. It is possible that as first-year medical students were exposed to the myriad components of what is needed to become a physician, their overall desire to use or conduct research in the future declined because they anticipated having far less time for research than originally thought. Further regular tracking of students’ intentions to use research in the future is needed to assess if these intentions will increase again later in medical training once students are more adjusted to the rigors of medical school. These findings also indicate that a clearer emphasis on “using research,” even when doing research in the future is not a goal, is needed and should be implemented within the MSRP.

### Change in Future Intent to Conduct Research

Data from the AAMC national surveys indicate that among medical students, research intentions (for “doing research”) overall tend to decrease between matriculation and MS2. In the AAMC national survey in 2022, 64.6% of students matriculating into medical school expressed interest in research careers (N = 15,061)(34), compared to 52.2% of MS2 students (N = 9,695)(35). Our data showed a drop in the percent of MSRP students intending to engage in research from 87.4% at baseline to 78.4% at the end of the first year of the program. Our results indicate that even with a drop in MSRP students’ interest in a future research after one year, compared to the AAMC data, a higher percentage of MSRP students at UCI were still interested in continuing to pursue research after the first year of the program (78% at one year among MSRP students compared to 52% nationally). Further, the proportion of MSRP students who intended to be significantly involved in research dropped from 56.8–45.9%. These changes should also be interpreted within the context of national AAMC data(37), showing that the general trend among first-year medical students is a decrease over time in their future research intentions. Nevertheless, the national data and our results remain reflective of the larger national problem of a “leaky pipeline” for physician scientists and provides evidence that this trend likely begins early in medical school(10).

### Possible Explanations for a Decline in Research Interest Among Students

The drop over time in medical students’ intentions to engage in research may be driven by several factors. It is possible that the driving motivation for the students to enroll in MSRP was to enhance the competitiveness of their residency application rather than an intrinsic interest in research. This explanation is in line with a survey study of medical students who were applying to plastic surgery residency which showed that the most common driving motivation to do research was to strengthen their credentials because of the residency competitiveness(38). However, in another study, personal interest in research was more commonly cited as a motivating factor in medical students who did two years of dedicated research compared to those who did one year of research(27). Since we only assessed the data at one year of follow-up, it is an open question whether intent to engage in future research among our MSRP participants changed at a later timepoint once students were more established in their research. It has been suggested that the decreased interest in research during the course of medical school may be attributed to the emphasis on clinical decision making rather than basic sciences(39). It is also likely that students gained a greater appreciation for the demands of a research career by the end of their first year of medical school, and the lower future intent to conduct research may reflect their perceptions of the complexity of balancing research with clinical practice and the added workload during medical school. One study of a six-month program showed the proportion of students who reported the medical student research program was stressful increased in the post-survey, because of the added workload on top of the existing curriculum(40). This may indicate students are interested in continuing with research in the future, but competing workloads may deter them from pursuing that path.

The mentor-mentee relationship is also an important consideration when discussing students’ intent to continue pursuing research. Previous studies have indicated that a good relationship with a mentor, positive team experiences, and experience with research training were critical factors in desire to do research(41, 42). Thus, greater insight into UCI MSRP students’ relationships with their mentors may be needed to understand the dynamics underlying this decline in research intention. There has been anecdotal evidence from our MSRP team of a trend among students to remain in a mentee-mentor relationship they view negatively long enough to produce an academic presentation or published manuscript, and then leave the research environment with no intent to continue with research. Future studies focusing on this type of experience would be needed to further parse out any role the mentee-mentor relationship plays in students’ desires to continue conducting research.

There are only a few programs similar to our MSRP that have reported the results of a pre and post-program survey(21, 40). A curriculum-based research program at the University of Texas Medical Branch at Galveston distributed a survey before and at the completion of the summer program between the first and second year of medical school(21). In the University of Texas study, the proportion of medical students who reported the intention to dedicate one-third to one-half of their career to research did not change significantly before and after the summer program (25.1% pre and 24% post program). In another program(40), although the proportion of students interested in future research increased after participation in a six-month research program, the change was non-significant and only increased by 3% (21.3–24.7%). Also of note, each of the above-mentioned programs are of short duration and it is unknown if the intent to pursue a research career will remain. In contrast, the MSRP is a long-term program and only one year of pre- and post-survey follow-up data is analyzed in this paper. To determine at what point students’ intent to use and conduct research declines and assess trends in whether research interest rebounds or consistently declines, further surveying at timepoints across medical school training are needed.

### Strengths of the Study

The program evaluation of the MSRP at UCI included tracking students from a baseline timepoint through the end of the first year of medical school. To date, only a few studies have been done with pre and post data for medical students involved in organized research programs to explore changes in their research skills and ongoing research interest. The MSRP assessments described in this paper provided longitudinal data for analysis with matched responses from program start to one year of completion, offering insight into how students’ perceptions and intentions changed after one year. As the students move through the program, additional timepoints of data at the students’ second, third, and final years of medical school will be used to explore trends over longer periods of time.

### Limitations

The major limitations of our program evaluation are the lack of a comparison group and participant attrition over time. In the absence of a comparison group, we caution against fully attributing our findings to the MSRP as an intervention. It is possible that the UCI medical school culture as a whole is supportive of research and that the MSRP is not the only avenue through which students can pursue their research interests. Indeed, we have noted anecdotally that some of the students who have discontinued their MSRP participation have done so because they independently connected with a mentor and no longer felt the need for the programmatic support to enable their research aspirations. We also note that our pre and post analysis included only those students who completed surveys at the one-year follow-up. As there were 59 students who failed to return one-year surveys, there is likely to be a strong impact of self-selection on the one-year scores. The students who responded to the follow-up survey might have reported strong research intentions because they successfully moved their research activities forward. We also note that our data only reflect the experience of medical students during their first year in the MSRP. The impact of the mentor-mentee relationship is more likely to emerge in the subsequent years of the program. Future analyses of this program will analyze the longer-term impacts of the program and examine the role of mentorship in students’ research intentions.

### Future Directions

Additional studies are needed to specifically assess if MSRP and similar programs at medical schools provide a meaningful buffer to the national problem of the “leaky” physician-scientist pipeline. The work reflected in this study does seem to indicate that participation in a research program increases research competencies, but the decline in students’ intent to use research in their clinical careers or conduct research in the future is still concerning. Incorporating responses from future medical student cohorts to increase the sample size, and adding a comparison group of non-MSRP enrolled participants could provide a clearer picture of MSRP’s impact and role in students’ motivations. Future analyses should also examine the longer-term impact of the program on future research intent and self-perceived knowledge gain by comparing second and third-year annual follow-up assessment results.

Previous data also suggest that high internal motivation to pursue research can predict students’ future involvement in a research-related career(43). Adding additional questions to student baseline and annual follow-up questionnaires related to internal motivation may also help us better understand how much the MSRP is contributing to retaining students in a research field as opposed to their own internal motivation regardless of support from MSRP. Additionally, collecting further qualitative data from medical students who experience a change over time in their intent to pursue future research may provide additional insight to further improve the MSRP to address the reasons students feel less inclined to pursue research. A comparison of this impact among students with different backgrounds and interests as well as varying research involvement during medical school also is needed.

Finally, future evaluations of medical student research programs might include tracking of student productivity in terms of research presentations and publications to illuminate the influence of this program on the physician-scientist workforce. Previous work has indicated that a higher degree of research output through publications and presentations during medical school leads to an increase in research during residency(27, 28), but future studies of UCI medical students could engage further longitudinal tracking of MSRP students into their residency training to determine what contributions are being made to the physician-scientist workforce after participation in a structured research program.

## Conclusions

In summary, although there are several limitations to our study, our program evaluation suggests that the University of California, Irvine Medical Student Research Program was successful in building students’ self-perceived competencies for engaging in research. The students may have also gained a realistic view of the complexities involved in doing research and although the proportion of students intending to engage in research decreased one year into the program, the majority of the students still expressed that they would like to be involved in research in the future. Providing a home for the students through ICTS is a most promising means to foster a robust infrastructure and an environment of improved collaboration and communication among the students and their mentors. Our curriculum-based program with support of faculty mentors in different specialties can be used as a model for other institutions to foster knowledge about research skills among medical students.

## Figures and Tables

**Table 1:** Medical Student Research Program Year 1 Curriculum

Introduction to Research
Conducting a Literature Review
Research Ethics
Institutional Review Board (IRB)
Informed Consent
Finding and Applying for Research Funding
Citation Management
Introduction to Research Statistics
Scientific Writing and Analyzing Publications
The Mentee-Mentor Relationship

**Table 2:** 12-Item Index of Perceived Research Competencies

Students were asked to rate themselves (on a five-point scale of No Confidence, Slight Confidence, Moderate Confidence, High Confidence, and Complete Confidence) on how much confidence they have at the current timepoint in their ability to do the following:
Evaluate the strength of a published research report on a new clinical practice
Locate relevant and high-quality published information on a new clinical practice
Derive translational questions from clinical research data
Design a research study protocol
Write up the results of a research project
Describe the concepts and implications of reliability and validity of study measurements
Describe the essential elements of voluntary informed consent
Describe the relevance of cultural and population diversity in clinical research design
Manage an interdisciplinary team of scientists
Communicate clinical and translational research findings to different groups of individuals, including colleagues, students, the lay public, and the media
Collaborate with bioinformatics specialists in the design, development, and implementation of research projects
Collaborate with biostatisticians in the design, conduct, and analyses of clinical and translational research

**Table 3:** Survey Items for Using Research in Clinical Practice Index

Students were asked to rate themselves (on a five-point scale of No Confidence, Slight Confidence, Moderate Confidence, High Confidence, and Complete Confidence) on how likely they think they are to do the following as part of their future clinical practice:
Keep up with the medical literature in my field
Continuously update my clinical practice in response to new research
Seek out new evidence-based approaches and treatments in my field
Inform my patients about their opportunities to participate in clinical trials
Partner with basic and/or pre-clinical researchers in research projects

**Table 4:** Mean at Baseline and Follow-up for Research Competencies and Intent for Using Research (N = 111)

	Baseline Mean (SD)	Follow-up Mean (SD)	p-value
Competencies Index	2.85 (0.73)	3.23 (0.56)	<0.001
Using Research Index	4.23 (0.61)	4.05 (0.67)	0.012

## Data Availability

The dataset supporting the conclusions of this article is available in the Dryad data repository [https://doi.org/10.5061/dryad.ns1rn8q24].

## Abbreviations

AAMC: Association of American Medical Colleges
CTSA: Clinical and Translational Science Award
IACUC: Institutional Animal Care and Use Committee
ICTS: Institute for Clinical and Translational Science
IRB: Institutional Review Board
LME: Liaison Committee on Medical Education
MS1: Medical Student Year 1
MS2: Medical Student Year 2
MSRP: Medical Student Research Program
REDCap: Research Electronic Data Capture
UCI: Univeristy of California, Irvine
USMLE: United States Medical Licensing Examination
